# Incidence Trend and Epidemiology of Common Cancers in the Center of Iran

**DOI:** 10.5539/gjhs.v8n3p146

**Published:** 2015-07-13

**Authors:** Hosein Rafiemanesh, Narjes Rajaei-Behbahani, Yousef Khani, Sayedehafagh Hosseini, Zahra pournamdar, Abdollah Mohammadian-Hafshejani, Shahin Soltani, Seyedeh Akram Hosseini, Salman Khazaei, Hamid Salehiniya

**Affiliations:** 1 School of Public Health, Tehran University of Medical Sciences, Tehran, Iran; 2 Department of Epidemiology and Biostatistics, Shahid Beheshti University of Medical Sciences, Tehran, Iran; 3 Alborz University of Medical Sciences, Karaj, Iran; 4 Endometriosis Research Center, Iran University of Medical Sciences, Tehran, Iran; 5 Pregnancy Health Research Center, Zahedan University of Medical Sciences, Zahedan, Iran; 6 Epidemiologist, School of Medicine, Rafsanjan University of Medical Sciences, Rafsanjan, Iran; 7 Department of Epidemiology and Biostatistics, School of Public Health, Isfahan University of Medical Sciences, Isfahan, Iran; 8 Department of Health Management and Economics, School of Public Health, Tehran University of Medical Sciences, Tehran, Iran; 9 Mashhad University of Medical Sciences, Nursing Collage of Quchan, Quchan, Iran; 10 Department of Epidemiology & Biostatistics, School of Public Health, Hamadan University of Medical Sciences, Hamadan, Iran; 11 Minimally Invasive Surgery Research Center, Iran University of Medical Sciences, Tehran, Iran; 12 Department of Epidemiology and Biostatistics, School of Public Health, Tehran University of Medical Sciences, Tehran, Iran

**Keywords:** incidence, cancer, trend, epidemiology, Iran

## Abstract

**Introduction:**

Cancer is a major public health problem in Iran and many other parts of the world. The cancer incidence is different in various countries and in country provinces. Geographical differences in the cancer incidence lead to be important to conduct an epidemiological study of the disease. This study aimed to investigate cancer epidemiology and trend in the province of Qom, located in center of Iran.

**Method:**

This is an analytical cross-sectional study carried out based on re-analysis cancer registry report and the disease management center of health ministry from 2004 to 2008 in the province of Qom. To describe incidence time trends, we carried out join point regression analysis using the software Join point Regression Program, Version 4.1.1.1.

**Results:**

There were 3,029 registered cases of cancer during 5 years studied. Sex ratio was 1.32 (male to female). Considering the frequency and mean standardized incidence, the most common cancer in women were breast, skin, colorectal, stomach, and esophagus, respectively while in men the most common cancers included skin, stomach, colorectal, bladder, and prostate, respectively. There was an increasing and significant trend, according to the annual percentage change (APC) equal to 8.08% (CI: 5.1-11.1) for all site cancer in women.

**Conclusion:**

The incidence trend of all cancers was increasing in this area. Hence, planning for identifying risk factors and performing programs for dealing with the disease are essential.

## 1. Introduction

Cancer is a major public health problem in Iran (Keyghobadi et al., 2015) and many other parts of the world. Approximately 14 million new cases of cancer and 8.2 million cancer deaths occurred worldwide. Based on GLOBOCAN in 2011, the Age-Standardized incidence Rate (ASR) of all cancers were105 and 165 per 100,000 person-years in men and women, respectively. Nearly 57% of new cancer cases and 65% of cancer deaths occurred in less developed regions (http://www.who.int/mediacentre/factsheets/fs297/en/, de Martel, 2012; Globocan, 2012; Malcolm, 2014; Negar Sadat Taheri et al., 2014).

The incidence of cancer is different in various countries. Ten common cancers in men in the world included lung, prostate, pancreas, lymph nodes, hematopoietic system, esophagus, stomach, bladder, kidney, and throat, but in women were lung, breast, pancreas, lymph nodes, hematopoietic system, stomach, cervix, kidney, rectum, and bladder (Joyce, 2007). According to the national report of cancer registry in 2009, 55.58% and 44.41% of cancer cases took place in Iran in men and women, respectively. Sex ratio was 1.25. As well, ten common cancers in Iranian men consisted of skin, stomach, prostate, bladder, colorectal, hematopoietic system, lung, esophagus, non-Hodgkin lymphoma, and brain and in women breast, skin, colorectal, stomach, esophagus, thyroid, hematopoietic system, ovary, brain, urinary tract (Iran Summer, 2011). Geographical differences in the cancer incidence lead to be important to conduct an epidemiological study of the disease. It is possible to find significant differences between developed and developing countries according to prevalence of cancers.

**Map 1:**
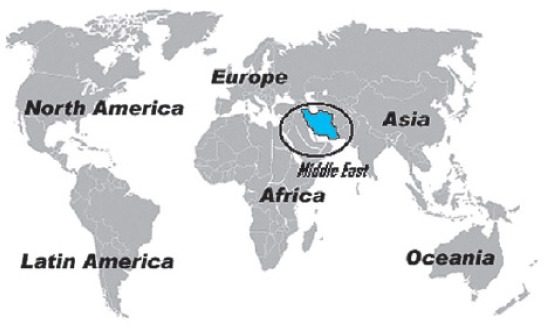
Iran location in the word

Iran is an ancient and developing countries country located in the Middle East, a region between Asia, Europe, and Africa. Iran is located in a special geostrategic situation by connecting the eastern and western parts of the world (Map 1). This country in recent years experiment the rapid development of modernity and industrialization, and changes in the environment and people's lifestyles, that this change may affect epidemiological patterns of different types of cancers (Elsayed et al., 2009; Abdolhassan Talaiezadeh et al., 2013; Marzieh et al., 2013; Almasi et al., 2015).

The area of Iran is 1,648,195 Km^2^ that makes it the 17th largest country in the world. Iran divided into 30 provinces and 336 districts. Iran with a population of almost 70 million, of which more than 65% are urban dwellers, Iran's population is young; almost one third of the population is less than 15 years old and only almost 5% is over 60 years. The population annual growth rate in 2006 was 1.2%. According to official data, more than 90% of Iranian people are under the coverage of at least one kind of health insurance. Cardiovascular diseases are the first cause of mortality in Iran with more than 45% of deaths; the second cause of mortality is accidents, with almost 18% of all deaths. The third causes of mortality were cancer with 14% of total deaths; so that 98 cases daily die from cancer in Iran (Iavari et al., 2006; Negar Sadat et al., 2014).

Qom is one of the provinces of Iran with 11,237 km², it is in the north of the country, and its provincial capital is the city of Qom. In 2011, this province had a population of 1,151,672 out of which 95.2% resided in urban areas and 4.8% in rural vicinities. The province contains 1 city, 4 counties, 9 rural districts, and 256 villages. There was a little information about the trend of cancer incidence in this provinces. Since the first step to dealing with the diseases and planning for the prevention, having knowledge of conditions and trends of cancer in any society, this study aimed to investigate cancer incidence in Qom between 2004 and 2008.

## 2. Methods

### 2.1 Study Design

This was an analytical cross-sectional study carried out based on re-analysis Cancer Registry Center report of health deputy which is based on Iran ministry of health guidelines, from 2004 to 2008 in the province of Qom in Iran.

### 2.2 Cancer Registry System in Iran

This analytic study was done based on longitudinal program in Iran that Similar to many countries in the world that have national registry of cancer (NCR) is trying to identify all cases of cancer occurring in Iran from 2004 to 2008. Data used in this study was obtained from a national registry of cancer (NCR), and Disease Control and Prevention (CDC) of ministry of Health and Medical Education in Iran for 2004 to 2008 (16). In 2008 in Iran, there are 30 provinces and 41 Medical Universities. Deputy for health of each university is responsible for health issues of the population and all health activities are managed by these deputies. All deputies for health have been included in the NCR. Registrar would apply the national registration software which was developed by CDC. For pathologic centers, without software, the cancer records were gathered manually. The Cancer Office of CDC should provide techniques and funding supports. The data are transmitted every 3 months, by electronic file and also hard copy of ‘Cancer Registry Data Collection Form’; this form is comprised of three parts: part I, regarding patient's identity characteristics in addition to the name of biopsy-taker physician, name of hospital, location of which the biopsy is taken, clinical diagnosis and date of biopsy sent to histological laboratory and demographic information of the patients includes race and residence. Part II includes the most important findings of patient's clinical history. Part III includes preclinical findings. The information includes primary location of tumor, date of cancer diagnosis, morphology and histology and its behavior and diagnosis method. Physicians fill the form of clinical data and the official personnel fill the identity and demographic information. Quality control has been coordinated in five main areas by Cancer Office of CDC: (i) regarding completeness of coverage; (ii) completeness of details; (iii) accuracy of data; (iv) accuracy of reports; (v) accuracy of interpretation and (vi) repeated cases are deleted from national data. Surveillance of pathology is based on the cancer record in several selected provinces to compare it with the present pathology cancer record for a general and complete evaluation and also for the accuracy of the collected data. IARC software provides a way to identify inaccuracies in data coding. Accordance of The International Classification of Diseases for Oncology (ICD-OC: topography with ICD-OM: morphology) is done manually and also by considering age and sex groups (pathology file of fatal error has been revised by the Scientific Society of Pathology of Iran and also by two masters in pathology).

Method for deleting repeated cases: for the lack of any classified National Identification Numbers, the process for deleting the repeated cases was completed by a manual review of the record. After editing data of each province and considering in mind that for deleting of repeated cases, similar cases should also be the same as morphology, topography, identity and demographic information; deletion of the repeated cases would be done separately in each province and finally in all over country by experienced manual reviewers.

### 2.3 Statistical Methods

The average annual age-standardized incidence rate (ASR) per 100 000 person-years was calculated by the direct method using the World Standard Population. To describe incidence time trends, we carried out join point regression analysis using the software Join point Regression Program, Version 4.1.1.1 October 2014. The analysis included logarithmic transformation of the rates, standard error, maximum number of one join points, and minimum of four years between zero join points. Like the least squares regression method, the joinpoint program is used to find the best-fit line through several years of data. However, the joinpoint program uses an algorithm that tests whether a multi-segmented line is a significantly better fit then a straight or less-segmented line. The test of significance uses a Monte Carlo Permutation method (i.e., it finds “the best fit” line). Like the least squares regression method, the joinpoint program is used to find the best-fit line through several years of data. Joinpoint regression analysis involves fitting a series of joined straight lines on a log scale to the trends. The aim of the approach is to identify possible joinpoints where a significant change in the trend occurs. In this study 0 joinpiont (Full model) was a significant model. All other program parameters were set to default values. All statistical tests were two sided. The significance level was considered as 0.05. Common cancers were defined as the number of reported cases and standardized incidence rates.

**Diagram 1:**
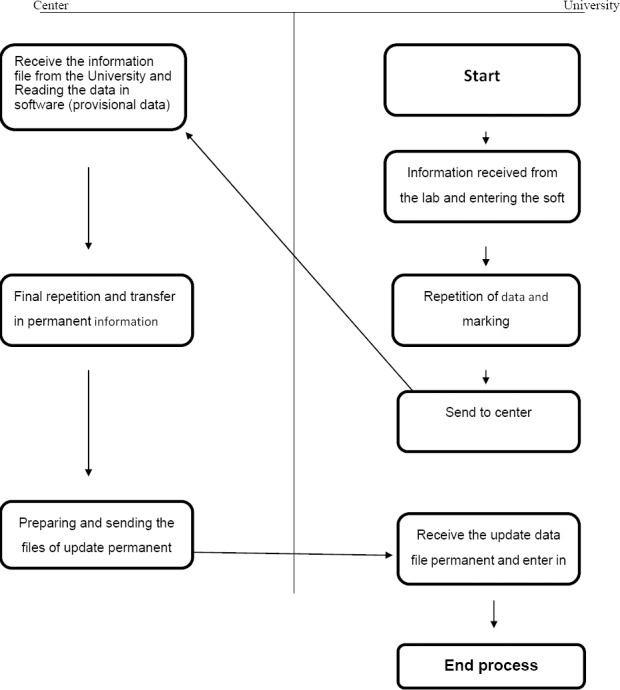
Process of data collection of cancer cause in Iran

## 3. Results

Overall, there was 3,029 registered cancer cases, of which 1383 case (43.1%) were women and 1826 cases (56.9%) men. Sex ratio was 1.32 (male to female). ASR was high in men than women. ASR was obtained about 70.09 per 100,000 people in women and 89.87 per 100,000 people in men. Considering mean standardized incidence, the most common cancer in women were breast, skin, colorectal, stomach and esophagus, while in men the most common cancers included skin, stomach, colorectal, bladder, and prostate. Five common cancers in men and women constituted 58.53% and 59.44%, respectively, from all cancers attributed to both sexes (Table 1).

**Table 1:** Frequency and standardized incidence of all sites and five common cancers by sex, during the years 2004 to 2008

				2004		2005		2006		2007		2008		Mean ASR	Total Count
**Sex**	Cancer			N	ASR	N	ASR	N	ASR	N	ASR	N	ASR	2004-2008	2004-2008
**Female**		All Site		212	66.69	256	67.74	279	74.84	298	81.53	338	89.67	76.09	1383
	Five Common Cancer	1	Breast	57	17.07	57	14.15	76	20.41	93	24.36	98	24.8	20.16	381
		2	Skin	30	11.34	34	9.63	26	8.08	35	10.63	34	9.07	9.75	159
		3	Colorectal	15	5.75	15	4.35	13	3.71	26	7.34	32	8.86	6.00	101
		4	Stomach	13	3.99	18	4.87	22	6.24	17	4.79	26	7.96	5.57	96
		5	Esophagus	15	4.94	18	4.99	15	4.29	20	6.28	17	4.49	5.00	85

	Total Five Cancer			130	43.09	142	37.99	152	42.73	191	53.4	207	55.18	46.48	822

**Male**		All Site		343	94.4	323	75.35	379	92.61	371	89.87	410	96.66	89.78	1826
	Five Common Cancer	1	Skin	62	17.79	57	14.43	57	14.32	49	13.01	61	14.19	14.75	286
		2	Stomach	42	12.30	58	13.64	63	15.86	59	14.36	58	13.87	14.01	280
		3	Colorectal	28	7.62	29	6.97	34	8.40	46	10.79	42	10.12	8.78	179
		4	Bladder	39	10.46	34	7.30	28	6.82	35	9.49	38	9.80	8.77	174
		5	Prostate	24	6.60	22	4.78	24	5.69	35	8.00	39	8.85	6.78	144

	Total Five Cancer			195	54.77	200	47.12	206	51.09	224	55.65	238	56.83	53.09	1063

## 4. Cancers in Women

There was an increasing and significant trend, according to the annual percentage change (APC) equal to 8.08% (CI: 5.1-11.1). The trend of five common cancers was 8.7% in women, which was not significant. Of five common cancers in women, breast, colorectal, and stomach increased, with APC of 13.8%, 1.3%, and 11.6%, respectively. Skin cancer (APC equal to -3.4%) had decreasing trend, while a constant trend was seen for esophagus cancer with APC of 0.4%). In above cancers, none of the trends in women showed significant changes. (p>0.05) (Table 2).

**Table 2:** Join point analysis for incidence of all site and five common cancer by sex in Qom, Iran, 2004-2008; ASR– age-standardized rate per 100 000 (using world standard population)

				2004 - 2008	

Sex	Cancer			APC	95% CI
**Female**		All Site		8.1^	5.1 to 11.1
	Five Common Cancer	1	Breast	13.8	-1.7 to 31.6
		2	Skin	-3.4	-16.1 to 11.2
		3	Colorectal	14.9	-17.4 to 59.9
		4	Stomach	14.6	-4.8 to 38.0
		5	Esophagus	0.4	-15.4 to 19.1

	Total Five Cancer			8.7	-2.0 to 20.6

**Male**		All Site		2.3	-8.2 to 13.9
	Five Common Cancer	1	Skin	-5.4	-13.3 to 3.2
		2	Stomach	3.0	-6.1 to 12.9
		3	Colorectal	10.6	-1.0 to 23.5
		4	Bladder	1.3	-18.7 to 26.3
		5	Prostate	11.6	-9.3 to 37.4

	Total Five Cancer			2.4	-5.2 to 10.7

^ APC and AAPC is significantly different from zero at alpha = 0.05.

**Cancers in men**

There was an increasing and significant trend, according to the annual percentage change (APC) equal to 2.3% (CI: -8.2-13.9). The trend of five common cancers was 2.4% in women, which was not significant. Of five common cancers in women, stomach, colorectal, bladder, and prostate increased, with APC of 3.8%, 10.6%, and 14.6%, respectively. Skin cancer (APC equal to -5.4%) had decreasing trend. In above cancers, none of the trends in women showed significant changes. (p>0.05) (Table 2).

**Figure 1:**
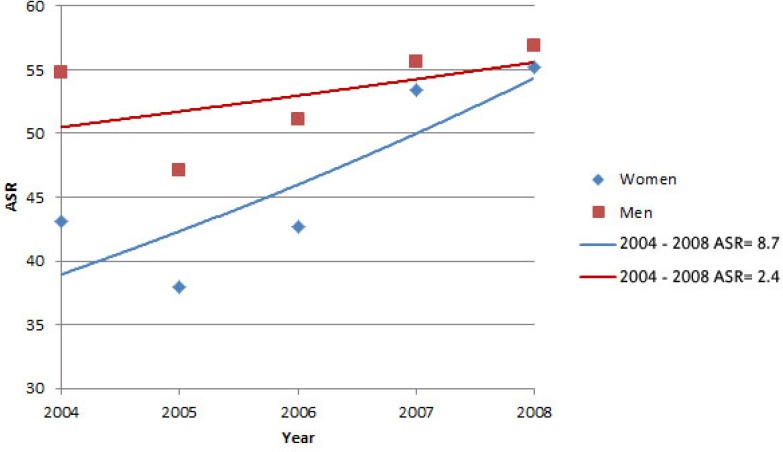
Overall trend of the five common cancers in men and women during 2004 to 2008 in Qom

## 4. Discussion

In this study, most patients were males (56.9%) and sex ratio was 1.32 (male to female). The prevalence of most of common cancers was high in men than women. Considering the mean standardized incidence, the most common cancer in women were breast, skin, colorectal, stomach and esophagus while in men the most common cancers included skin, stomach, colorectal, bladder and prostate, respectively. There was an increasing and significant trend, according to the annual percentage change (APC) equal to 8.08% (CI: 5.1-11.1) for all site cancer in women.

Based on the national report of cancer registry in 2009, most of patients were men (55.58%). The sex ratio of cancer incidence in the country was 125 (MoHotIRo, Summer 2011) and similar to other studies (JM, 1991; S. M. Jalali & S. A. K. I. Jalali, 2005; Samareh Pahlavan, 2006), male patients were high. Maisinneuve and Lowenfels reported that the prevalence of cancer was twice in men than women (Lowenfels, 2006). Studies performed in Belgium in 2003 (Buntinx, 2003), Canada and the United States (Asulin, 2004), and Western Europe (Black et al., 1997) indicted consistent findings, but the opposite occurred in Eastern Europe, except French (Black et al., 1997).

In our study, ASR was 89.87 per 100,000 in men and 76.09 per 100,000 in women. Based on the national report of cancer registry in 2009, ASR was 132.19 in men and 123.55 in women (MoHotIRo, Summer 2011). Cancer incidence in Europe was 446 in men and 284 in women, but the incidence was 303 in men and 204 in women per 100,000 people in the world (Buntinx et al., 2003), while the incidence was lower in Mediterranean countries (Trichopoulou et al., 2000). In Ardebil (the region in North West of Iran), cancer incidence was 132 per 100,000 in men and 96 per 100,000 in women (Sajadi & Derakhshan, 2003). In Polynesia, located in French, the incidence was 186 and 209 in men and women, respectively. The incidence was 132.6 and 133 in Pakistan in men and women, respectively (Gleize, 2000; Bhurgri & Hassan, 2002). ASR was 153 and 156 per 100,000 people in Khuzestan (the province in Western South of Iran) in men and women, respectively (Neda Amoori & Maria, 2014). ASR for all cancers was 164.3 and 130.9 in Western Azerbaijan, located in North West of Iran, in men and women, respectively(Mohammad, 2008). Therefore, the incidence of cancer in Iran, was lower than some areas in the word. It seems that the cancer incidence estimated in Iran wasn’t real, and in dead we under estimate the incidence and mortality of cancer in Iran, due to problems such as The Low Quality of cancer registry System in Iran and this fact that in many cases we don’t biopsy and therefore undiagnosed of disease in this case. However, national data in Iran, show an increasing trend in incidence and mortality of cancer (Mohagheghi et al., 2009). In contrast to the situation, in certain developed countries in last decade the incidence and mortality of cancers were decreasing trend (Mousavi et al., 2007; Jemal et al., 2010; Bray et al., 2012). This difference might be due to fast changes in lifestyle, exposure to risk factors, aggregation of carcinogens, and air and environment pollution in Iran that led to increasing trend in cancer incidence.

The incidence of cancer is different in various regions. Five common cancers in men in the world are: lung, prostate, colorectal, stomach, and liver and in women, including breast, colorectal, lung, cervical, and stomach (Organization, 2012). In the country (Iran), five common cancers in men are skin, stomach, prostate, bladder, and colorectal cancer, while in women, breast, skin, colorectal, stomach, and esophagus (MoHotIRo., Summer 2011). Based on the national report of cancer registry in 2009, five common cancers in men were skin, stomach, colorectal, bladder, and lung, while in women, breast, colorectal, skin, stomach, and esophagus in Qom (MoHotIRo., Summer 2011). A study, conducted in East Azerbaijan, showed that five common cancer in men included stomach, skin, bladder, esophagus, and colorectal and in women breast, skin, esophagus, stomach, and colorectal (Mohammad et al., 2008). A study in Khuzestan indicated that the most common cancers in men comprised skin, stomach, lung, blood, prostate, and bladder and in women, breast, skin, colorectal, lung, and bladder (Neda Amoori & Maria, 2014).

In our study, five common cancers in men were skin, stomach, colorectal, bladder, and prostate and in women, breast, skin, colorectal, stomach and esophagus. There was a small difference between our results and national reports, but a considerable difference between them and world reports. The differences in the incidence of cancer should be investigated to perform identifying related risk factors. Other studies also expressed high incidence of stomach cancer in men and breast cancer in women. However, the differences in incidence of lung and colorectal cancers between our findings, and national and world statistics can relate to lifestyles and features of the area. Therefore, it is necessary to identification risk factors associated with these cancers and developing screening program. According to the high prevalence of stomach cancer in the country and Qom, it is required to endoscopy and detailed examination for individuals at risk. In recent decades, breast cancer is the first common cancer in women in the country and world, and is increasing. Other studies emphasized an increasing trend of the cancer (Afsoon Taghavi et al., 2012; Aboulfazl Afsharfard et al., 2013; Mitra Rahimzadeh, Mahmood, & Mohamad, 2014; Neda Amoori & Maria, 2014).

Our findings, similar to other studies (Krittika Suwanrungruang et al., 2006; Seyed et al., 2010; Edris Abdifard et al., 2013; Nikbakht Roya, 2013), showed that colorectal cancer is enhancing in both sexes and the trend is high in men than women. This may be due to dietary habits and changes in life style. Several studies indicated that high-fat diet, obesity, tobacco use, and lack of physical activity are risk factors for this cancer. Since colorectal cancer is the preventable disease, it is necessary to more investigate about risk factors affecting the cancer in Qom.

In our study, the incidence of bladder cancer was increasing in men so that the cancer was one of the common cancers in males in this region. Some studies also showed that the incidence of cancer is higher in men than women (Alireza Salehi et al., 2011; Mohammad Ahmadi et al., 2012). This may be due to high consumption of tobacco and Opium, and exposure to occupational carcinogens in men than women.

Our results revealed that the incidence of stomach cancer is increasing in both sex, so in 2004-2008 was the second cancer in men and Third in women. Another study stated that the incidence of the stomach cancer have Increasing trend (Krittika Suwanrungruang et al., 2006). In the study, carried out in Khuzestan (one province of Iran), higher incidence of the cancer was seen in women than men (Neda Amoori & Maria, 2014). Risk factors attributed to the cancer are Helicobacter pylori, genetics, diet, and environmental factors. Hence, endoscopy and accurate examinations are essential for detection individuals at the risk factors.

## 5. Limitations

There were some limitations in our study. Data in our registry were limited to pathology, residency, sex, and age while other related variables like feeding pattern, job, and other lifestyle and socioeconomic factors have an important role in susceptibility to cancer. Additionally registration in that period of time was limited to the pathology system so a large number of cancers were missed. it should be stated that the cancer registry system in Iran is still not fully and equally in all area and sometimes the differences in the quality and coverage of data is observed.

## 6. Conclusion

It can conclude that the incidence of all cancer is increasing in this area. Therefore, the plan for the control and prevention of this disease must be a high priority for health policy makers. Our findings was obtained from the descriptive study on the incidence trend of the disease in recent years and it is recommended that analytical studies should be conducted to obtain a causal relationship and solve problems related to the disease.
